# Endoscopic lithotripsy of a gallstone impacted in lumen-apposing metal stent positioned for cholecysto-gastrostomy

**DOI:** 10.1016/j.vgie.2023.03.008

**Published:** 2023-06-17

**Authors:** Tommaso Gabbani, Giuliano Francesco Bonura, Paolo Biancheri, Paola Soriani, Mauro Manno

**Affiliations:** Gastroenterology and Digestive Endoscopy Unit, Azienda USL Modena, Carpi, Italy

## Abstract

Video 1 Endoscopic lithotripsy of a gallstone impacted in the lumen-apposing metal stent positioned for cholecysto-gastrostomy.

Endoscopic lithotripsy of a gallstone impacted in the lumen-apposing metal stent positioned for cholecysto-gastrostomy.

## Introduction

EUS-guided gallbladder drainage with a lumen-apposing metal stent (LAMS) is a viable treatment option for acute cholecystitis; it is an alternative to cholecystectomy in patients at high risk for surgery, with fewer adverse events than percutaneous drainage.^1^ However, recurrent cholecystitis with stone impaction is a possible adverse event in patients with large calculi.^2^ We report the case of a patient with acute recurrent lithiasic cholecystitis who was deemed unfit for surgery owing to her age and multiple comorbidities and was then treated with cholecysto-gastrostomy.

## Case Presentation

An 85-year-old woman was admitted to our unit with sepsis from acute cholecystitis. She was deemed unfit for surgery because of her age, comorbidities (chronic kidney disease and congestive heart failure), and overall clinical conditions. Thus, transgastric echoendoscopic gallbladder drainage was performed, using a 15- × 10-mm LAMS (Hot Axios; Boston Scientific, Natick, Mass, USA), with subsequent clinical improvement. A double-pigtail plastic stent was not inserted immediately after LAMS insertion to minimize the risk of LAMS displacement.

After 3 months, the patient came back to our attention with recurrent acute cholecystitis and sepsis (Video 1, available online at www.videogie.org).

**Figure 1:**
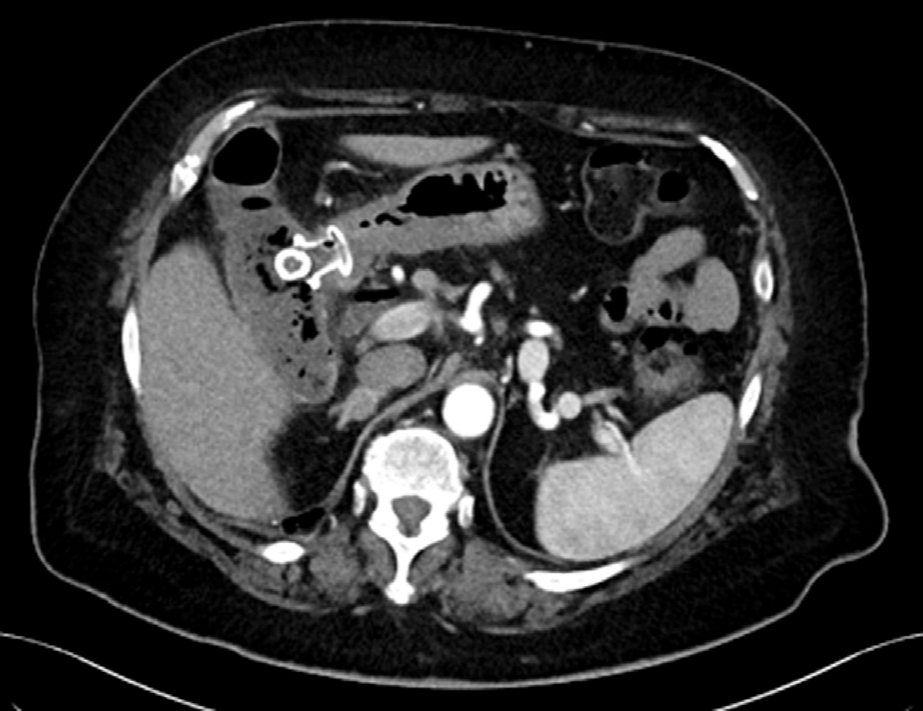
CT scan image of a 15-mm stone impacted in the distal flange of the lumen-apposing metal stent.

**Figure 2:**
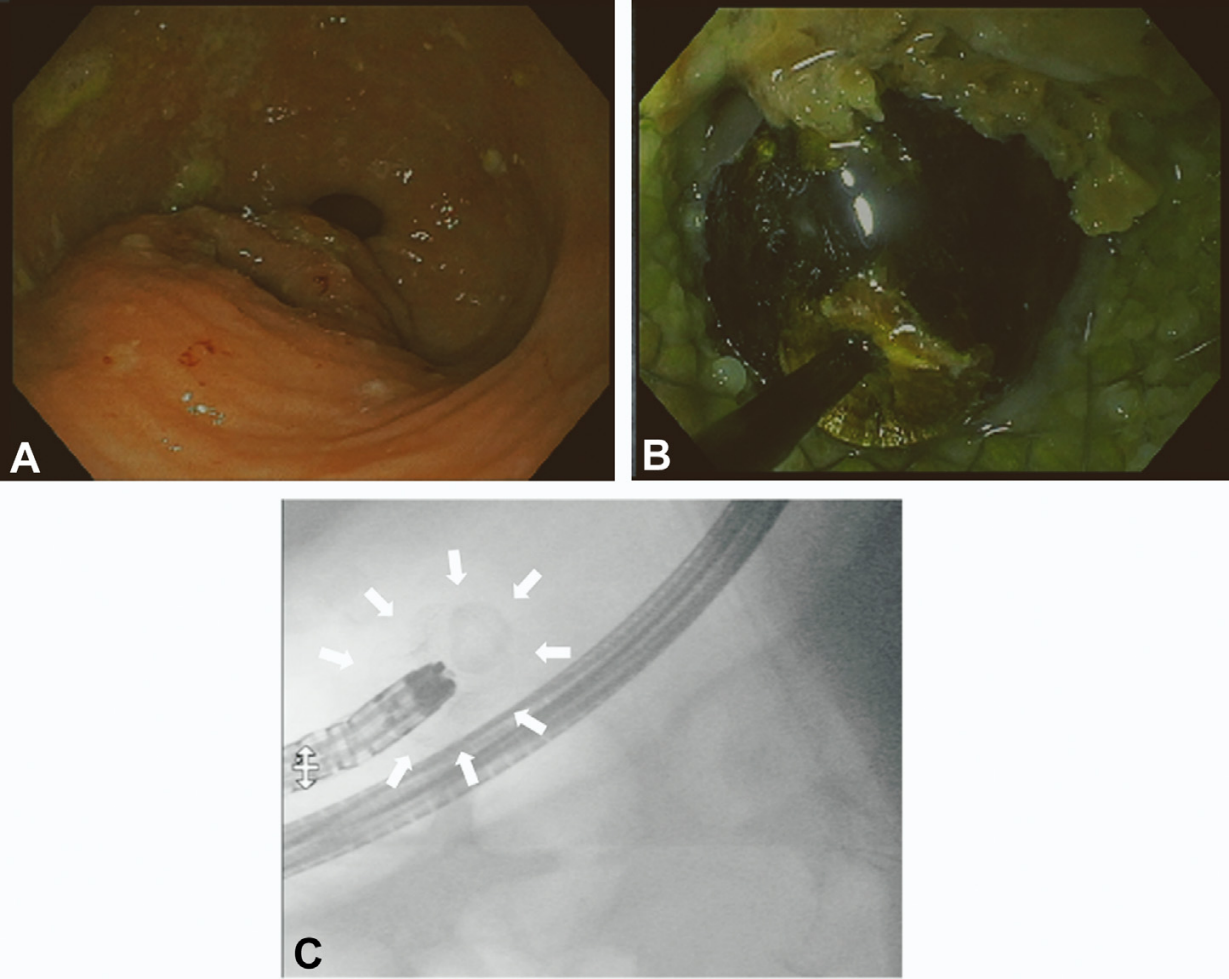
Endoscopic view of partially buried lumen-apposing metal stent **(A)** with the lumen obstructed by an impacted biliary stone **(B)**, confirmed by fluoroscopic imaging **(C)**.

**Figure 3:**
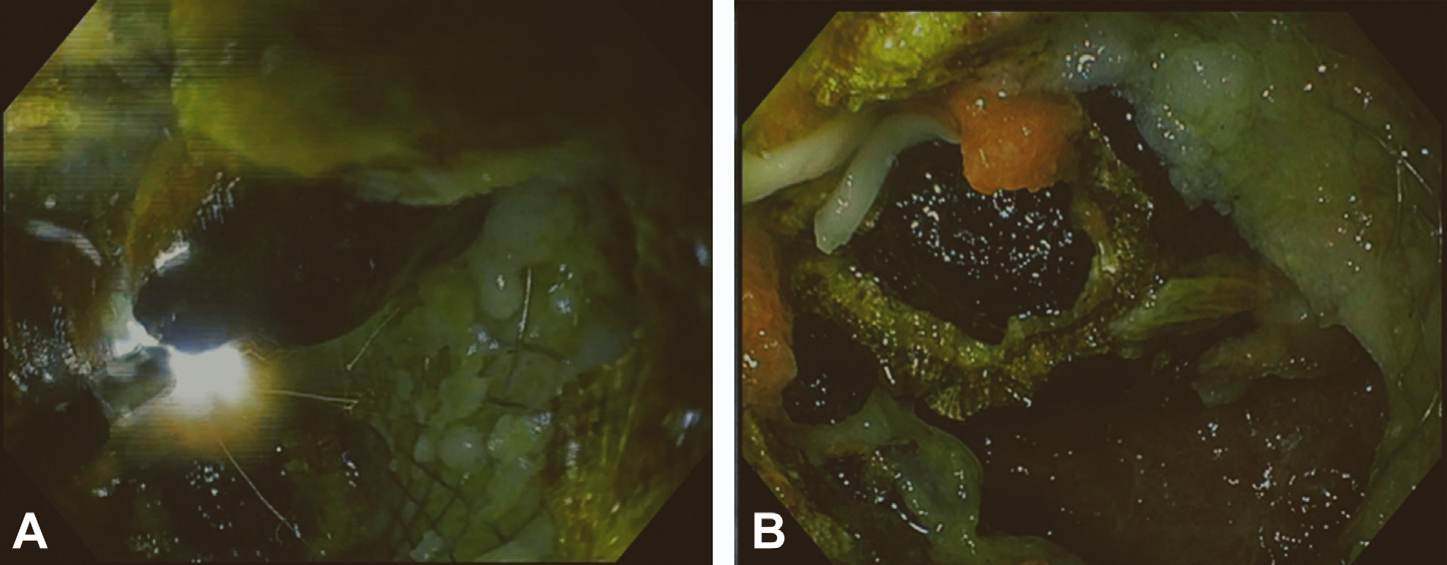
**A,** Endoscopic electrohydraulic lithotripsy with partial fragmentation of the stone. **B,** Subsequent drainage of debris and large amounts of pus.

**Figure 4:**
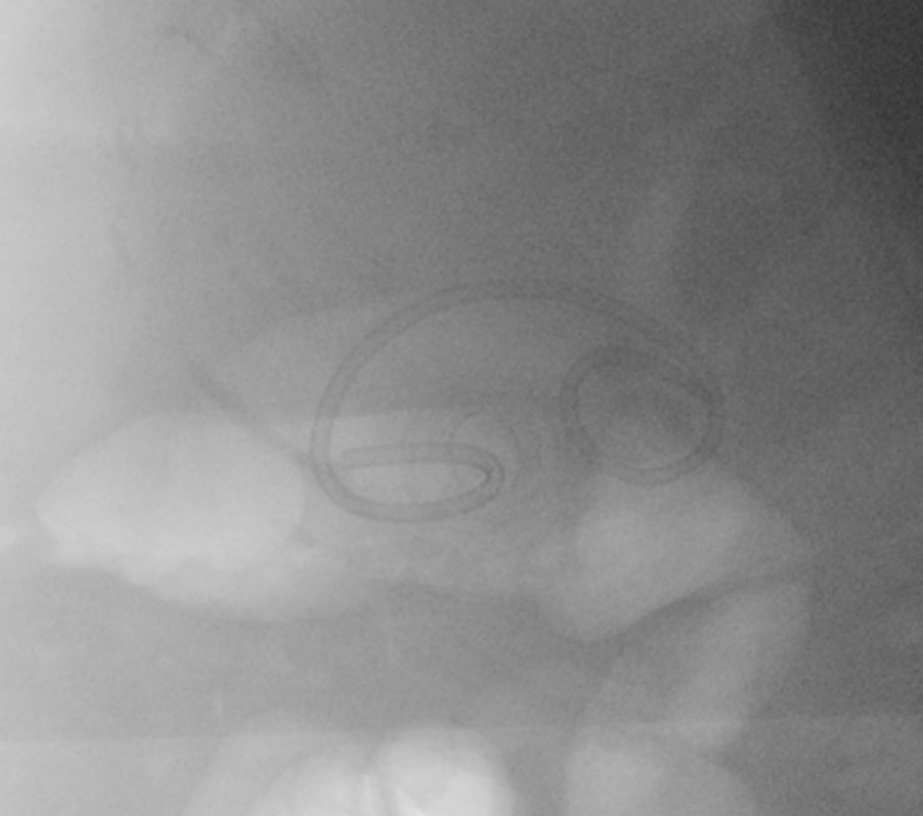
Fluoroscopic view of double-pigtail plastic stent placed through the lumen-apposing metal stent.

A CT scan showed a 15-mm stone impacted in the distal flange of the LAMS (Fig. 1). Upper endoscopy (GIF-1TH190; Olympus, Tokyo, Japan) showed a partially buried LAMS with the lumen obstructed by an impacted biliary stone (Fig. 2). Attempts to remove or to push the stone into the gallbladder failed. Thus, endoscopic electrohydraulic lithotripsy (EEHL) (1.9F, 375-cm Biliary EEHL Probe Autolith; Boston Scientific) was performed. The EEHL probe was introduced through the endoscope, and fragmentation upon infusion of saline solution was started. Lithotripsy was performed starting at 15 pulses and low power, and it was progressively increased up to 30 pulses and high power because of the hard core of the stone. Resolution of the stone impaction by partial fragmentation of the stone was achieved, with subsequent drainage of debris and large amounts of pus (Fig. 3). Larger stone fragments were removed with a retrieval basket, and most of the small stone fragments were removed by pump washing. Finally, using a duodenoscope (TJF-Q190V; Olympus), a 10F double-pigtail plastic stent was placed through the LAMS to prevent further obstruction of the cholecysto-gastrostomy (Fig. 4). No intra- or periprocedural adverse events were observed, and 3 days later the patient was discharged home as asymptomatic. Considering the severe comorbidities and the frail clinical conditions of this elderly patient, which made her unfit for surgery, we decided to keep both the LAMS and the double-pigtail stent in place permanently. At her 6-month follow-up evaluation, the patient remained asymptomatic with normal biochemistry.

## Conclusions

EUS-guided gallbladder drainage in patients deemed unfit for surgery is an effective and safe technique. Rarely, stone impaction and LAMS obstruction can occur.^2^^,^^3^ In this scenario, EEHL is a useful technique that, by generating high-amplitude hydraulic pressure waves with repetitive pulses of energy to create a mechanical shockwave,^4^^,^^5^ allows fragmentation of the impacted stone. EEHL may be a good option to treat LAMS obstruction in cholecysto-gastrostomy.

## Disclosure

*The authors disclosed no financial relationships.*

## Supplementary data

Video 1 Endoscopic lithotripsy of a gallstone impacted in the lumen-apposing metal stent positioned for cholecysto-gastrostomy.
